# The Enigmatic Protein Kinase C-eta

**DOI:** 10.3390/cancers11020214

**Published:** 2019-02-13

**Authors:** Alakananda Basu

**Affiliations:** Department of Microbiology, Immunology & Genetics, University of North Texas Health Science Center, Fort Worth, TX 76107, USA; Alakananda.basu@unthsc.edu; Tel.: +1-817-735-2487

**Keywords:** protein kinase C, PKCη, cell proliferation, differentiation, senescence, apoptosis, drug resistance, tumor promotion, tumor suppression

## Abstract

Protein kinase C (PKC), a multi-gene family, plays critical roles in signal transduction and cell regulation. Protein kinase C-eta (PKCη) is a unique member of the PKC family since its regulation is distinct from other PKC isozymes. PKCη was shown to regulate cell proliferation, differentiation and cell death. It was also shown to contribute to chemoresistance in several cancers. PKCη has been associated with several cancers, including renal cell carcinoma, glioblastoma, breast cancer, non-small cell lung cancer, and acute myeloid leukemia. However, mice lacking PKCη were more susceptible to tumor formation in a two-stage carcinogenesis model, and it is downregulated in hepatocellular carcinoma. Thus, the role of PKCη in cancer remains controversial. The purpose of this review article is to discuss how PKCη regulates various cellular processes that may contribute to its contrasting roles in cancer.

## 1. Introduction

Intricate regulation of cellular signaling systems is critical for the proper functioning of cells. Consequently, a deregulation in signal transduction pathways can lead to many human diseases. Protein kinase C (PKC), a family of serine/threonine protein kinases, plays critical roles in signal transduction and cell regulation [1]. The identification of PKC as a receptor for tumor-promoting phorbol esters, which are potent activators of PKC and can substitute for the physiologic stimulator diacylglycerol (DAG) established a link between PKC and cancer [2].

PKC constitutes a multi-gene family that could be categorized into three groups based on their structural variations and biochemical properties [3]. The classical or conventional (c) PKCs (α, βI, βII, γ) require Ca^2+^ and DAG/phorbol esters for their activities. The novel (n) (δ, ε, η, θ) PKCs are insensitive to Ca^2+^ but respond to DAG/phorbol esters. The atypical (a) PKCs (ξ, ι) are insensitive to both Ca^2+^ and DAG/phorbol esters. While the physiological stimulator DAG causes transient activation of conventional and novel PKCs, the tumor-promoting phorbol esters cause persistent activation [3]. Activation of PKCs induces their translocation to the membrane followed by their degradation or downregulation [4]. 

The regulation of PKCη, a member of the novel PKC family, is unique [3]. Although PKCη is most closely related to PKCε [5,6], there are variations in the lipid-binding site [7]. It is the only PKC that is activated by cholesterol sulfate and sulfatide [8]. It is also resistant to translocation and downregulation when stimulated with phorbol esters or cholesterol sulfate [9,10]. In fact, we and others have shown that PKCη is upregulated by the tumor-promoting phorbol esters [11,12,13,14] as well as several structurally and functionally distinct PKC activators [13].

The expression of PKCη is also unique compared to other PKC isozymes [15,16]. It was isolated from cDNA libraries of mouse epidermis [5] and human keratinocytes [15]. PKCη mRNA was most abundant in lung tissues and was also detected in skin and heart tissues [15]. Contrary to other PKC isozymes, the expression of PKCη in the brain was low [15,16]. It was shown to be predominantly expressed in the epidermis of mouse skin and epithelia of the digestive and respiratory tracts, including the tongue, esophagus, forestomach, glandular stomach, intestine, colon, trachea and, bronchus [16].

**Figure 1:**
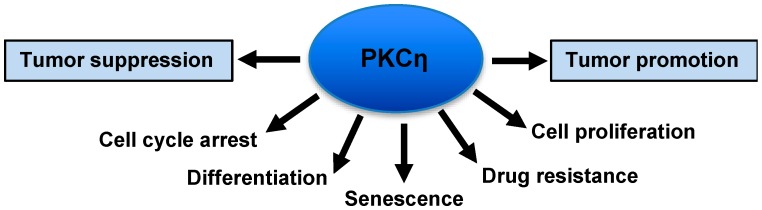
Various functions of protein kinase C-eta (PKCη).

PKCη plays critical roles in cell proliferation, differentiation and cell death, as seen in Figure 1 [3,8,17]. There are, however, controversies regarding its role in tumor promotion versus tumor suppression. The present review article summarizes how PKCη regulates various cellular processes that may impact on its contrasting roles in cancer.

## 2. Regulation of Cell Proliferation

The most consistent function of PKCη is its ability to regulate cell cycle progression. The cell cycle is regulated primarily by the tumor suppressor protein Rb and cyclin-dependent kinases (CDK). While phosphorylation and inactivation of Rb by CDKs is needed to allow cell cycle progression, the inhibition of Rb phosphorylation by CDK inhibitors, such as p16, p21 and p27 causes cell cycle arrest [18].

Interestingly, both activation and inhibition of PKCη have been reported to cause cell cycle arrest (Table 1). Livneh et al. first reported that ectopic expression of PKCη in NIH3T3 cells inhibits Rb phosphorylation and induces CDK inhibitors p21 and p27, causing cell cycle arrest [19]. Overexpression of PKCη also inhibited cell growth in keratinocytes but in this study, PKCη overexpression had no effect on cell growth in either human or mouse fibroblasts [20]. On the other hand, Nomoto et al. showed that overexpression of PKCη in NIH3T3 cells induced anchorage-independent growth [21]. The reason for the distinct effects of PKCη overexpression on cell growth in NIH3T3 fibroblasts is not clear except different methods were used to monitor fibroblast cell growth. For example, while Livneh et al. [19] examined how PKCη affects cell cycle progression by analyzing different phases of the cell cycle by flow cytometric analysis, Ohba et al. [20] monitored cell growth by MTT assay, and Nomoto et al. [21] compared the colony forming ability of NIH3T3 cells transfected with either PKCη or a control vector in soft agar.

Kashiwagi et al. provided a mechanistic explanation that association of PKCη with cyclin E/Cdk2/p21 complex causes phosphorylation of p21 at Ser146 site and dephosphorylation of Thr160 of Cdk2 resulting in inhibition of Cdk2 activity and G1 arrest in keratinocytes [8,22]. Subsequently, Livneh and co-workers confirmed that PKCη also forms complexes with cyclin E/Cdk2 in NIH3T3 and MCF-7 cells, and this complex formation was most prominent in serum-starved cells and could be visualized in the perinuclear region [23]. However, PKCη overexpression had opposite effects on cell growth in NIH3T3 versus MCF-7 cells. In contrast to NIH3T3 cells where PKCη overexpression was shown to inhibit cell growth [19], induced expression of PKCη in MCF-7 cells promoted cell growth [24]. Overexpression of PKCη caused an increase in both p21 and p27 in NIH3T3 cells [19] but p27 was not altered in MCF-7 cells [24]. Thus, an increase in p27 may be required for cell growth inhibition in MCF-7 cells. Consistent with this notion, we recently observed that knockdown of PKCη decreased clonogenic survival of breast cancer MCF-7 and T47D cells and this was associated with an increase in p27 [25]. Hara et al. showed that p27 mRNA was downregulated in PKCη-null keratinocytes grown in 3D organotypic culture [26]. While an increase in p27 was associated with cell growth inhibition in both keratinocytes [26] and breast cancer cells [25], depletion of PKCη caused a decrease in p27 in keratinocytes [26] but an increase in p27 in breast cancer cells [25]. It is not clear why PKCη had opposite effects on p27 in keratinocytes versus breast cancer cells, however, it reinforces the notion that PKCη functions in a context-dependent manner.

An increase in PKCη was shown to be responsible for the anti-leukemic effects of IFNα in chronic myeloid leukemia cells [27]. PKCη overexpression caused cell cycle arrest in normal and leukemic human myeloid cells but had no effect on erythroid progenitor cells [27]. PKCη was shown to promote cell proliferation in glioblastoma cells by acting upstream of Akt and mTOR signaling pathways [28]. Knockdown of PKCη inhibited cell cycle progression in B lymphoma cells, suggesting a growth-promoting effect of PKCη [29]. Thus, the function of PKCη on cell proliferation varies significantly with cell types (Table 1).

## 3. Regulation of Differentiation

Most of the earlier studies associated growth inhibitory effects of PKCη with its ability to trigger differentiation partly because PKCη was most abundant in epithelial tissues [46], was expressed during epidermal differentiation [47] and was shown to be localized in differentiating or differentiated epithelial cells [16]. Moreover, cholesterol sulfate, a metabolite of cholesterol generated during squamous differentiation caused marked stimulation of PKCη activity [48]. PKCη levels were increased both at the soluble and particulate fractions of primary mouse keratinocytes during calcium-induced differentiation [49].

Several studies investigated potential mechanisms by which PKCη triggers keratinocyte differentiation. Ohba et al. [20] demonstrated that overexpression of PKCη in human and mouse keratinocytes but not in fibroblasts enhanced the expression and activity of transglutaminase 1, a key enzyme involved in squamous cell differentiation. PKCη was shown to associate with and activate the Src kinase family member Fyn, which is required for normal keratinocyte differentiation [32]. Overexpression of Fyn enhanced the expression of CDK inhibitors p21 and p27, induced the differentiation marker transglutaminase, and suppressed the growth of keratinocytes but had no effect in dermal fibroblasts [32]. These results provided an explanation why PKCη induced differentiation in keratinocytes but not in fibroblasts. PKCη also increased JunD-mediated transcription of loricrin, which is expressed at the late stage of keratinocyte differentiation [30]. The small G protein RalA was shown to interact with PKCη resulting in the activation of RalA and the induction of keratinocyte differentiation [31]. Hara et al. [26] demonstrated that growth arrest and terminal differentiation were delayed in PKCη-null keratinocytes and this was associated with downregulation of p27 mRNA via c-Jun N-terminal kinase (JNK) signaling. Re-expression of PKCη or suppression of JNK/c-Jun signaling caused upregulation of p27 mRNA resulting in cell cycle arrest and terminal differentiation [26]. Overexpression of the C2 domain of PKCη increased the expression of collagen type II, and led to chondrogenic differentiation in mesenchymal stem cells [45]. Thus, an association between inhibition of cell proliferation and differentiation was established.

## 4. Regulation of Apoptosis

Apoptosis is a physiologic form of cell death required to maintain tissue homeostasis. Lack of cell death by apoptosis can lead to cancer. In addition, since many cancer chemotherapeutic drugs kill cancer cells by inducing apoptosis, a defect in apoptotic signaling pathways can contribute to chemoresistance. There are two major pathways of cell death: the extrinsic or receptor-initiated pathway and the intrinsic or mitochondrial pathway. The extrinsic pathway is triggered by binding of ligands to the tumor necrosis factor-α (TNF) receptor superfamily, whereas cytotoxic chemotherapeutic drugs primarily utilize the intrinsic pathway. While activation of caspases induces apoptosis, pro- and anti-apoptotic Bcl-2 family members regulate apoptosis.

We observed that several different PKC activators protected against TNF-induced apoptosis whereas the PKC-specific inhibitor bisindolylmaleimide II enhanced apoptosis in breast cancer MCF-7 cells [11]. Since PKCη is the only PKC isozyme upregulated by PKC activators and downregulated by the PKC inhibitor bisindolylmaleimide II, this study implicated PKCη in protecting against TNF-induced apoptosis [4]. Beck et al. showed that there was a correlation between the upregulation of multiple drug resistance-associated genes and PKCη expression in specimens derived from sixty-four primary breast cancer patients, implicating PKCη in anticancer drug resistance [50]. While these results are correlative, we showed that ectopic expression of PKCη in MCF-7 cells protected against TNF-induced apoptosis [36]. Subsequently, it was shown that PKCη protects against apoptosis induced by UV and gamma irradiation in glioblastoma cell lines [40]. Downregulation of PKCη by anti-sense oligonucleotides (ODN) enhanced vincristine- and paclitaxel-induced apoptosis in A549 lung cancer cells [43] and TNF-related apoptosis-inducing ligand (TRAIL)-induced apoptosis in prostate cancer PC3 cells [44]. PKCη levels were higher in Hodgkin’s lymphoma (HL)-derived L428 cells that are resistant to doxorubicin and camptothecin (CPT) compared to drug-sensitive KMH2 cells, and knockdown of PKCη by siRNA in L428 cells sensitized them to these drugs [42]. In addition, PKCη suppressed and shR-PKCη promoted cisplatin-induced apoptosis in adenoid cystic carcinoma (ACC) cells [34]. These results suggest that PKCη may confer resistance to anticancer therapy.

Several studies explored the mechanisms by which PKCη regulates apoptosis. Overexpression of wild-type PKCη inhibited and dominant-negative PKCη enhanced UVB-induced apoptosis in normal human keratinocytes (NHK) [33]. UV-induced activation of p38 MAP kinase suppressed caspase-3 activity in NHK, and this was blocked by dominant-negative PKCη, suggesting that PKCη negatively regulates UV-induced apoptosis in NHK cells via the p38 MAP kinase pathway [33]. In MCF-7 breast cancer cells, PKCη was shown to protect against DNA damaging agents, such as UVC irradiation and anticancer drug CPT by suppressing JNK activity [37]. PKCη also protected against CPT via activation of NF-κB, leading to the induction of anti-apoptotic Bcl-2 in MCF-7 cells [38].

Akt, mTOR and mitogen-activated protein kinase (MAPK) pathways are known to promote cell survival. There are, however, controversies regarding how PKCη regulates the Akt signaling pathway. While Aeder et al. reported that PKCη activates both Akt and mTOR pathways in glioblastoma cells [28], Shahaf et al. reported that PKCη negatively regulates Akt in MCF-7 cells [51]. In the latter study, Akt activation was monitored in response to IGF-1 or insulin stimulation [51].

Development of resistance to the BCR-ABL inhibitor imatinib mesylate (IM) is a significant problem in the treatment of chronic myelogenous leukemia (CML). Upregulation of *PRKCH*, the gene encoding PKCη, was identified as one mechanism contributing to IM resistance independent of any mutation in BCR-ABL [41]. *PRKCH* was elevated in IM-resistant CML patient samples and CML stem cells [41]. The mechanism by which PKCη contributed to IM resistance involved activation of the RAF/mitogen-activated protein kinase (MAPK)/extracellular signal-regulated kinase (ERK) signaling via phosphorylation/activation of CRAF [41]. We have recently shown that knockdown of PKCη in breast cancer cells led to the downregulation of the anti-apoptotic Bcl-2 family protein Mcl-1 via the ubiquitin proteasome-mediated pathway [52]. Knockdown of PKCη inhibited ERK1/2 phosphorylation but knockdown of ERK1, but not ERK2, decreased Mcl-1 levels in MCF-7 cells. Moreover, overexpression of ERK1 rescued the effect of PKCη knockdown on Mcl-1 downregulation [52]. These results suggest that PKCη functions upstream of ERK1 in MCF-7 breast cancer cells.

## 5. Regulation of Senescence

Cellular senescence is defined as a permanent arrest of proliferative cells that are metabolically active [53]. The consequences of senescence could be beneficial or detrimental depending on the cellular context, the nature of the stimulus and the state of senescence [54,55,56]. Senescence can cause tumor suppression by inducing permanent cell cycle arrest and by recruiting immune systems to clear senescent cells [57,58]. However, senescent cells can also contribute to tumor progression and relapse. Senescence-associated secretory phenotype (SASP), which is associated with the secretion of growth factors, pro-inflammatory cytokines, chemokines, and matrix remodeling enzymes, could facilitate tumor growth under certain cellular contexts [57,59].

Zurgil et al. reported that PKCη promotes senescence in MCF-7 breast cancer cells in response to oxidative stress and etoposide-induced DNA damage [35]. In contrast, we found that knockdown of PKCη induced senescence in breast cancer MCF-7 and T47D cells [25]. The apparent discordant results could be partly explained by the differences in experimental design. In the study by Zurgil et al., high concentrations of H_2_O_2_ (150 µM) or etoposide (400 µM) caused a substantial increase in senescence, which was attenuated by PKCη knockdown [35]. In fact, knockdown of PKCη by itself caused a modest but significant increase in cellular senescence [35], and this was consistent with our results [25]. shRNA-mediated knockdown of PKCη had little effect on p27 and p21 but attenuated the increase in p21 and p27 by etoposide [35]. In addition, PKCη knockdown increased IL-6 secretion but suppressed IL-8 secretion [35]. It is not clear why PKCη had opposite effects on these pro-inflammatory cytokines both of which are associated with SASP. We found that silencing of PKCη by siRNA caused a substantial increase in p27 in both MCF-7 and T47D cells [25]. Moreover, silencing of p27 attenuated senescence induced by PKCη knockdown [25], suggesting upregulation of p27 as one mechanism contributing to the induction of senescence caused by PKCη deficiency.

## 6. Tumor Suppression by PKCη

Canzian et al. first reported that PKCη is decreased by 5- to 10-fold in murine lung tumors compared to normal murine lung [60], suggesting that a decrease in PKCη may be associated with lung carcinogenesis. A clue to the tumor suppressive role of PKCη came from the observation that cholesterol sulfate, which acts as a second messenger of PKCη and induced squamous differentiation, inhibited skin carcinogenesis when applied prior to tumor-promoting phorbol ester TPA. This suggests that PKCη inhibits the promotional phase of skin carcinogenesis [61]. Further evidence regarding the tumor suppressive role of PKCη came from the observation that PKCη-knockout mice were more sensitive to tumor formation in a two-stage carcinogenesis model compared to wild-type mice [62]. The ability of PKCη to inhibit tumor promotion was associated with its ability to induce differentiation in keratinocytes [8].

The possible tumor suppressive role of PKCη was also investigated by analyzing human tissue samples. PKCη mRNA was significantly lower in colon tumors compared to normal mucosa samples [63]. PKCη expression was decreased in locally invasive breast tumor tissues compared to the surrounding normal epithelium, suggesting that PKCη is decreased during later stages of transformation [64]. Overexpression of PKCη was shown to exert anti-leukemic responses in chronic myeloid leukemia cells by inhibiting cell cycle progression of myeloid progenitor growth via type 1 interferon receptor [27]. PKCη expression was decreased in 82% of hepatocellular carcinoma (HCC) tissues compared to adjacent normal tissues and was associated with poorer long-term survival of HCC patients [65].

## 7. Tumor Promotion by PKCη

Overexpression of PKCη in NIH3T3 cells was shown to induce anchorage-independent growth [21], suggesting that PKCη may also contribute to tumor progression. A correlation between PKCη expression and tumor progression was noted in renal cell carcinoma (RCC) [66]. PKCη was shown to promote proliferation of malignant astrocytoma and glioblastoma but not non-malignant astrocytes [28,39]. PKCη levels were high in EBV-transformed B cells and EBV-positive B cells, such as Raji, Daudi and IM-9 cells but not in normal B cells [29]. We have shown that PKCη levels were increased with the aggressiveness of breast cancer in the progressive MCF-10A series, and knockdown of PKCη attenuated breast cancer cell growth [25,67]. Increased PKCη expression was associated with poor prognosis in non-small cell lung cancer patients [68]. It has been reported that the microRNA (miRNA) miR-24-3p functions as a tumor suppressor in human lacrimal adenoid cystic carcinoma (LACC) via the p53/p21 pathway by downregulating PKCη, and overexpression of PKCη rescued the tumor suppressive function of miR-24-3p by downregulating p53 [34]. PKCη levels were higher in LACC tissues compared to adjacent non-tumor tissues and overexpression of miR-24-3p decreased PKCη mRNA and protein levels [34]. PKCη contributed to the malignant phenotype of ACC cells by enhancing cell proliferation, migration and invasion and by inhibiting apoptosis [34]. The *PRKCH* gene was highly expressed in hematopoietic stem cells (HSC) and leukemia stem cells (LSC) [69]. PKCη expression was also associated with poor prognosis in acute myeloid leukemia (AML) patients, although it was not required for the development of AML [69].

## 8. Conclusions

The function of PKCη varies significantly with cell types (Table 1). Several laboratories showed that PKCη induced terminal differentiation in keratinocytes [8,20,26,31,32] and PKCη-null mice were more susceptible to tumor formation [61,62]. In contrast, there were controversies regarding the role of PKCη in NIH3T3 fibroblasts [19,20,21]. The transcriptional regulation of PKCη [70] as well as the regulation of downstream signaling of PKCη [32] appear to be distinct in keratinocytes versus fibroblasts.

Caution should be exercised in interpreting the function of PKCη in various systems. Some of the studies that implicated PKCη in tumor promotion versus tumor suppression are correlative, and the number of tumor samples analyzed to definitively assess the function of PKCη may not be adequate. In studies involving genetic manipulation of PKCη, it is important to consider the methods used to manipulate PKCη, the selection pressure and the extent of PKCη knockdown/overexpression, all of which may affect the outcome of the results. The specificity of the antibodies used to detect total and phosphorylated PKCη should also be carefully determined.

It is also important to recognize the complexity of the cellular signaling systems. For example, a decrease in cell proliferation may also promote epithelial-to-mesenchymal transition and is associated with increased malignancy [71]. This may explain why overexpression of PKCη inhibited cell growth [19] but enhanced anchorage-independent growth [21] in NIH3T3 cells. Similarly, a decrease in cell growth is often associated with an increase in cell death by apoptosis. However, inhibition of cell proliferation may also restrict the ability of conventional chemotherapeutic drugs that target actively proliferating cells to kill cancer cells. This is consistent with our observation that the induction of senescence caused by PKCη knockdown was associated with a decrease in doxorubicin-induced apoptosis [25]. In addition, while overexpression of PKCη inhibited cell growth in NHK [8,20,26,31,32], PKCη overexpression inhibited UVB-induced apoptosis in these cells [33].

PKCη interacts with several signaling pathways and the status of these pathways will influence the function of PKCη. The ability of PKCη to regulate cellular senescence may also contribute to its contrasting roles in cancer since depending on the cellular context, cellular senescence may promote or suppress cancer. Thus, a thorough understanding of how PKCη regulates various cellular processes is essential prior to exploiting this enigmatic PKC family member for cancer therapy.

## Figures and Tables

**Table 1:** Function of PKCη in different cell/tumor types.

Tissue/Tumor Type	Cell Line	Expression	Phenotype	Potential Mechanism	Reference
Fibroblasts	NIH3T3	WT-PKCη	Cell cycle arrest, adipogenesis	↑ p21, 27 & cyclin E; ↓ Rb phosphorylation	[19]
	NIH3T3	WT-PKCη	Anchorage-independent growth		[21]
	NIH3T3 & NHF	WT-PKCη	No effect on cell growth		[20]
Keratinocytes	NHK	WT-PKCη	Growth arrest, terminal differentiation	↑ p21 Phosphorylation; ↓ Cdk2, ↑ TGase 1	[20,22]
	NHK	WT-PKCη	Differentiation	↑ Loricrin	[30]
	NHEK	WT-PKCη	Differentiation	Bidning and activation of RalA	[31]
	Mouse keratinocytes	WT-PKCη	Growth arrest, differentiation	↓ EGFR, ↑Fyn activity	[32]
		PKCη-null	Delayed growth arrest & terminal differentiation	↑ JNK/cJun, ↓ p27	[26]
	NHK	DN-PKCη	Inrease in UVB-induced apoptosis	↓ UV-induced p38 MAPK activity	[33]
Adenoid cystic carcinoma	ACC-2 & ACC-M	WT-PKCη	Suppressed cisplatin-induced apoptosis	↓ p53/p21	[34]
Breast cancer	MCF-7	WT-PKCη	Increase in cell growth	↑ cyclin D, -E and p21	[24]
	MCF-7, T47D	PKCη siRNA	Decreased clonogenic survival, induced senescence	↑ p27	[25]
	MCF-7	PKCη shRNA	Decreased H2O2 and etoposide-induced senescence	↓ p21, p27 & IL-6; ↑ IL-8	[35]
	MCF-7	WT-PKCη	Protects against TNF-induced apoptosis		[36]
	MCF-7	WT-PKCη	Protects against UVC- and CPT-induced apoptosis	↓ JNK activity	[37]
	MCF-7	WT-PKCη	Protects against CPT-induced apoptosis	↑ NF-κB activity	[38]
Glioblastoma	U-251 GBM	PKCη-KR	Decreased cell proliferation	↓ Akt, mTOR activity	[28]
	U-1242 MG	WT-PKCη	Icreased cell proliferation	↑ ERK/Elk-1 activity	[39]
	U-1242 MG	WT-PKCη	Decrease in UV- and Υ irradiation-induced apoptosis		[40]
	U-251 MG	PKCη-antisense	Sensitized to UV- and Υ irradiation-induced apoptosis		[40]
Leukemia	CML-derived KT1	Peptide inhibitor	Suppression of IFN-dependent cell cycle arrest		[27]
	CD34+ progenitor	PKCη siRNA	Increase in clonogenic survival		[27]
	CD34+ progenitor	CA-PKCη	Inhibition of cell growth, no effect on apoptosis		[27]
	CML K562	WT-PKCη	IM resistance	↑ Raf/MEK/ERK signaling and CRAF	[41]
	CML stem cells	PKCη shRNA	Sensitized to IM		[41]
Lymphoma	IM-9, EBV+ B cells	PKCη siRNA	Cell cycle arrest	↑ TAp73, p21, p38 MAPK; ↓ Cdks	[29]
	IM9	PKCη siRNA	Sensitized to BTZ and SRF		[29]
	L428	PKCη shRNA	Sensitized to doxorubicin- and CPT-induced apoptosis		[42]
Lung cancer	A549	PKCη-antisense	Increased vincristine and paclitaxel-induced apoptosis		[43]
Prostate cancer	PC3	PKCη-antisense	Increased TRAIL-induced apoptosis		[44]
Mesenchymal stem cells	hMSC	PKCη-C2	chondrogenic differentiation	Increase in collagen type II	[45]

## Abbreviations

**Abbrev.**	**Definition**
ACC	adenoid cystic carcinoma
AML	acute myelocytic leukemia
Bcl-2	B-cell lymphoma 2
Btz	bortezomib
CDK	cyclin-dependent kinase
CML	chronic myelogenous leukemia
CPT	camptothecin
DAG	diacylglycerol
DN	dominant-negative
EGFR	epidermal growth factor receptor
ERK	extracellular signal-regulated kinase
GBM	glioblastoma multiforme
HSC	hematopoietic stem cells
IFN	Interferon-α
IM	imatinib mesylate
JNK	c-Jun N-terminal kinase
LSC	leukemia stem cells
MAPK	mitogen-activated protein kinase
Mcl-1	myeloid cell leukemia 1
mTOR	mechanistic target of rapamycin
MTT	3-(4,5-dimethylthiazol-2-yl)-2,5-diphenyltetrazolium bromide
NF-κB	nuclear factor-κB
NHEK	normal human epidermal keratinocytes
NHF	normal human fibroblasts
NHK	normal human keratinocytes
ODN	oligonucleotide
PKC	protein kinase C
SASP	senescence-associated secretory phenotype
SRF	Sorafenib
TNF	tumor necrosis factor-α
TGase 1	transglutaminase 1
TPA	12-*O*-tetradecanoylphorbol-13-acetate
TRAIL	TNF-related apoptosis-inducing ligand
WT	wild-type
